# Methylglyoxal-induced glycation stress promotes aortic stiffening: putative mechanistic roles of oxidative stress and cellular senescence

**DOI:** 10.18632/aging.206335

**Published:** 2025-11-14

**Authors:** Parminder Singh, Ravinandan Venkatasubramanian, Sophia A. Mahoney, Mary A. Darrah, Katelyn R. Ludwig, Alice Zhang, Kiyomi Kaneshiro, Lizbeth Enriquez Najera, Lauren Wimer, Muniesh M. Shanmugam, Edgard Morazan, James J. Galligan, Marrisa N. Trujillo, Richmond Sarpong, Douglas R. Seals, Pankaj Kapahi, Zachary S. Clayton

**Affiliations:** 1 Buck Institute for Research on Aging, Novato, CA 94945, USA; 2 University of Colorado Boulder, Boulder, CO 80309, USA; 3 College of Chemistry, University of Berkely, Berkely, CA 94720, USA; 4 R. Ken Coit College of Pharmacy, University of Arizona, Tucson, AZ 85721, USA; 5 University of Colorado Anschutz Medical Campus, Aurora, CO 80045, USA

**Keywords:** glycation stress, methylglyoxal, pulse wave velocity, gly-low, vascular dysfunction

## Abstract

Background: Here, we assessed the role of the advanced glycation end-product (AGE) precursor methylglyoxal (MGO) and its non-crosslinking AGE MGO-derived hydroimidazolone (MGH)-1 in aortic stiffening and explored the potential of a glycation stress-lowering compound (Gly-Low) to mitigate these effects.

Methods: Young (3–6 month) C57BL/6J mice were supplemented with MGO (in water) and Gly-Low (in chow). Aortic stiffness was assessed *in vivo* via pulse wave velocity (PWV) and *ex vivo* through elastic modulus. Putative mechanisms underlying MGO- and MGH-1-induced aortic stiffening were explored using complementary experimental approaches in aortic tissue and cultured human aortic endothelial cells (HAECs). Moreover, aortic stiffness was assessed in old C57BL/6J (24 month) mice after consumption of Gly-Low-enriched chow.

Results: MGO-induced glycation stress increased PWV in young mice by 21% (P<0.05 vs. control), which was prevented with Gly-Low (P=0.93 vs. control). Ex vivo, MGO increased aortic elastic modulus ~100% (P<0.05), superoxide production by ~40% (P<0.05), and MGH-1 expression by 50% (P<0.05), which were all mitigated by Gly-Low. Chronic MGO exposure elevated biomarkers of cellular senescence in HAECs, comparable to a known senescence inducer Doxorubicin, an effect partially blocked by Gly-Low. Moreover, elevated aortic elastic modulus induced by Doxorubicin (P<0.05 vs. control) was prevented with Gly-Low (P=0.71 vs. control). Aortic RNA sequencing implicated preservation of endogenous cellular detoxification pathways with Gly-Low following exposure to MGH-1. Old mice supplemented with Gly-Low had lower PWV (P<0.05) relative to old control mice.

Conclusions: MGO-induced glycation stress contributes to aortic stiffening and glycation stress lowering compounds hold promise for mitigating these effects.

## INTRODUCTION

Cardiovascular diseases (CVD) remain the leading cause of mortality worldwide, with elevated aortic stiffness being a significant predictor of CV-related morbidity and mortality [1]. Advancing age is the primary non-modifiable risk factor for the development of CVD. A key event mediating the increase in CVD risk with aging is stiffening of the large elastic arteries (primarily the aorta) [2].

Age-related aortic stiffening, as demonstrated by increased aortic pulse wave velocity (PWV), occurs in part via increased accumulation of advanced glycation end products (AGEs), which results from non-enzymatic glycation of proteins and lipids [3, 4]. Despite the biomedical importance of AGEs, the underlying molecular mechanisms contributing to AGEs-mediated aortic stiffening with aging are not fully understood. Notably, AGEs can accumulate in multiple layers of the arterial wall, including the intima, media, and adventitia, and can adversely affect vascular compliance [5].

Methylglyoxal (MGO), a reactive α-dicarbonyl compound, is a key potent precursor of AGEs and is significantly elevated in arteries in old age [6]. The accumulation of MGO-derived AGEs has been implicated in various pathophysiological processes [6, 7]. To capture these combined effects, we use the term glycation stress, which we define as the cumulative burden of reactive carbonyls such as MGO and their AGE adducts, which drive structural and functional impairments in tissues.

MGO preferentially reacts with arginine (Arg) residues to form specific AGEs, such as MGO-derived hydroimidazolone isomer (MGH-1), an established marker of MGO-induced glycation stress [8]. This interaction is particularly relevant to vascular function because Arg is the amino acid substrate for endothelial nitric oxide synthase (eNOS), which produces the vasoprotective molecule nitric oxide (NO) [9]. Thus, MGO-mediated modifications of Arg residues may limit NO bioavailability, contributing to further vascular dysfunction and increased stiffness. Elevated circulating concentrations of MGH-1 are strongly related to CVD risk and CV-related mortality in individuals with type 2 diabetes [10–12]. Although MGH-1 has been identified as a hallmark of MGO-induced glycation stress, its specific role in driving the pathological processes underlying aortic stiffening—including oxidative stress and cellular senescence—remains incompletely understood.

In the present study, we used a series of complementary *in vivo*, *ex vivo*, and *in vitro* experimental approaches to determine the causal role of MGO-induced glycation stress in aortic stiffening and the putative underlying mechanisms mediating this response, including excessive oxidative stress and cellular senescence. Additionally, we explored the therapeutic potential of Gly-Low, a cocktail consisting of the natural compounds nicotinamide, pyridoxine, thiamine, piperine, and alpha-lipoic acid [13], in mitigating aortic stiffening, oxidative stress and cellular senescence mediated by MGO-induced glycation stress.

We hypothesized that MGO-induced glycation stress is a key driver of age-related aortic stiffening through the induction of oxidative stress and cellular senescence. Moreover, we posited that reducing MGO accumulation and its downstream AGEs with Gly-Low would attenuate these pathological processes, thereby preserving vascular compliance and preventing aortic stiffening.

## MATERIALS AND METHODS

### Study design and experimental animals

Young male and female C57BL/6J mice were acquired from Jackson laboratory and aged male and female C57BL/6J were acquired from the colony of the National Institute of Aging (maintained by Charles River, Wilmington, MA, USA). Prior to initiating the study, mice were given 4 weeks to acclimate to the animal housing facility. Mice were group-housed with a 12-h:12-h light:dark cycle and given ad libitum access to their respective rodent chow*.* young mice were 3–6 months at the beginning of experiment and interventions lasted for 8 weeks; old C57BL/6J mice were 20–24 months at the beginning of experiment and interventions lasted for 16 weeks. For the intervention, mice received 1% MGO in drinking water. For the Gly-Low intervention mice were fed either a modified AIN-93G diet (21% fat (kcal), 60% carbohydrate (kcal) Envigo: TD.200743), or modified AIN-93G diet supplemented with our Gly-Low compound cocktail (Envigo: TD.200742). A combination of supplemental grade compounds, safe to be consumed in the administered dosages, were prepared and incorporated into a modified pre-irradiated diet. The cocktail consists of alpha lipoic acid (20.19%) (Sigma-62320), nicotinamide (57.68%) (Sigma-72340), thiamine hydrochloride (4.04%) (Sigma-T4625), piperine (1.73%) (Sigma-T49007), and pyridoxamine dihydrochloride (16.36%) (Sigma-P9380). To achieve the desired daily intake in mg/kg body weight/day, the cocktail mixture was incorporated into the diet at a concentration of 14.86 g/kg of diet. Consequently, the final concentrations of individual components in the 1X Gly-Low diet were calculated as follows: 3 g/kg alpha lipoic acid, 8.57 g/kg nicotinamide, 0.6 g/kg thiamine hydrochloride, 0.26 g/kg piperine, and 2.43 g/kg pyridoxamine dihydrochloride. These values were derived using the formula: (Percentage in cocktail / 100) × total cocktail added per kg of diet. Drinking water was replaced every 48 hours and we did not observe any significant differences between the control and MGO-treated groups.

### *In vivo* aortic stiffness (PWV)

Aortic stiffness was assessed using the reference standard non-invasive *in vivo* measure, aortic pulse wave velocity (PWV), one week after the intervention, as previously described [9, 14]. Briefly, male and female mice were placed under light isoflurane anesthesia (1.0%–2.5%) delivered in 100% oxygen at a flow rate of 1 L/min and positioned supine on a warmed heat pad. Front- and hind-limb paws were then secured to corresponding ECG electrodes. Two Doppler probes were then placed on the skin at the transverse aortic arch and the abdominal aorta. Once clear R-waves were registered, three repeated 2-second ultrasound tracings were recorded and average pre-ejection time (i.e., time between the R-wave of the ECG to the foot of the Doppler signal) was determined for each location. To calculate aortic PWV, the distance between the two probes was divided by the difference between the transverse aortic arch and abdominal aorta pre-ejection times (time_abdominal_ – time_arch_) and is reported as centimeters/second (cm/s).

### Sacrifice and tissue collection

Mice were euthanized using a method approved under the American Veterinary Medical Association guidelines. Mice were anesthetized under inhaled anesthesia (open-drop method) and euthanized via cardiac exsanguination. The aorta was excised and rinsed in physiological saline solution (PSS), cleared of perivascular adipose and connective tissue, sectioned, and stored as described below.

### *Ex vivo* aortic intrinsic mechanical wall stiffness (elastic modulus)

Two 1mm segments of thoracic aorta were collected at sacrifice from intervention-naïve male C57BL/6 mice. Then, aortic rings from each donor animal were incubated in the following conditions in duplicate for 48 hours. The control condition was DMEM + 1% penicillin-streptomycin (Standard media). For other agents used, their concentrations were 1uM TEMPOL (Sigma-Aldrich, Cat. No. 2226-96-2), 500uM MGO (Sigma Aldrich, Cat #M0252), 100uM Gly-Low, 200nM MGH-1(made as described previously [13]), 100uM Glo-1 inhibitor (Sigma Aldrich, Cat #SML1306), and 1uM DOXO (R&D Systems, cat #2252/50). After the incubation period, elastic modulus was assessed using the pin myograph (Danish Myo Technology, Denmark) as previously described [15]. Briefly, 1mm aorta rings were mounted on to two prongs in a phosphate-buffered saline (PBS) bath heated to 37° C. After three rounds of pre-stretching (pins displaced to 1mm), the aortic diameter was increased until a force of 1mN was reached and then was incrementally increased by 50μM every three minutes until mechanical failure. The force corresponding to each stretching interval was recorded and utilized to calculate stress and strain and to construct a stress-strain curve:

Strain (λ) = Δd/d_i_
Where d is diameter and d_i_ is the initial diameter.
Stress(t) = (λL)/2(HD)
Where L is one-dimensional load, H is intima-media thickness, and D is vessel length.

The elastic modulus of the stress-strain curve was determined as the slope of the linear regression fit of the final four points of the stress-strain curve before the aortic rings broke as previously reported by our laboratory [15]. Aortic wall thickness and diameter were assessed in 1mm aortic rings frozen in optimal cutting temperature, which were stored in -80C until the time of sectioning. Aortic sectioning was carried out in a cryostat (Leica Biosystems, Wetzlar, Germany) at - 22°C. 7um sections were visualized and imaged under brightfield microscope and ImageJ was used to quantify the aortic thickness and diameter. Changes in aortic elastic modulus were presented as a fold-change relative to the control condition.

### Aortic ROS levels

Whole-cell aortic ROS production was assessed by electron paramagnetic resonance (EPR) spectrometry using the spin probe 1-hydroxy-3-methoxycarbonyl-2,2,5,5-tetramethylpyrrolidine (CMH; Enzo Life Sciences, Farmingdale, NY, USA, Cat. No. ALX-430-078), as previously described [15]. In short, two 1-mm aortic rings were washed in warm physiological saline solution and incubated in Krebs/HEPES buffer, consisting of 99 mM NaCl, 4.7 mM KCl, 1.87 mM CaCl2, 1.2 mM MgSO4, 25 mM NaHCO3, 1.03 mM KH2PO4, 20 mM Na-HEPES, 11.1 mM glucose, 0.1 mM diethylenetriaminepenta-acetic acid, 0.0035 mM sodium diethyldithiocarbamate, and Chelex (Sigma-Aldrich, Cat. No. C7901), containing 0.5 mM CMH at 37° C for 60min. Samples were analyzed using aMS300 Xband EPR spectrometer (Magnettech, Berlin, Germany) with the following instrument parameters: B0-Field, 3350G; sweep, 80G; sweep time, 60s; modulation, 3000mG; MWatten, 7dB; gain, 500.

### Aortic immunofluorescence

Immunofluorescence assay was performed to visualize the subcellular localization of a prominent MGO byproduct methylglyoxal derived hydroimidazolone-1 (MGH-1). After the 48-hour incubation period mentioned in the subsection “*Ex vivo* aortic intrinsic mechanical wall stiffness” ~1mm section of thoracic aorta was excised and frozen in OCT (Tissue-Tek^®^ O.C.T.) compound, as described previously [15]. Following, 7 μm sections (Leica CM1520) were plated on poly-L-lysine-coated microscope slides, fixed in 4% paraformaldehyde, washed with PBS, and permeabilized (0.1% Triton X-100). Slides were then stained with anti-MGH-1 primary antibody (1:200; Cell Biolabs, San Diego, CA, USA; Cat# STA-011) according to instructions from the mouse-on-mouse immunodetection kit (Vector Laboratories, Newark, CA, USA; Cat# BMK-2202), and then incubated with a species-specific fluorescent secondary antibody (1:400, Alexa Fluor 555; Invitrogen, Waltham, MA, USA) for 30 minutes. Slides were washed, stained with DAPI (1:1000; Invitrogen, Waltham, MA, USA; Cat# D1306) for 5 minutes, and cured overnight with ProLong Gold mounting media (Invitrogen, Waltham, MA, USA; Cat# P36980). These slides were then imaged using EVOS m7000 (Thermo Fisher Scientific, Waltham, MA, USA; Cat# AMF7000) fluorescence microscope under identical conditions and analyzed using Invitrogen Celleste 5.0 Image Analysis Software. Images were obtained at 10x magnification. The abundance of MGH-1 was determined as the average intensity (in arbitrary units A.U.) of the positively stained area across N = 6-8 samples/animal. Specificity of the MGH-1 antibody was determined using negative controls (secondary antibody only condition) in a subset of samples.

### HAEC RNA extraction and cDNA synthesis

mRNA gene expression was measured in HAECs following mechanical homogenization. RNA was extracted using the RNeasy mini kit (Qiagen, Hilden, Germany). cDNA was synthesized using the iScript cDNA synthesis kit (Bio-Rad Laboratories, Hercules, CA, USA). Transcripts of cellular senescence-related genes (primers reported in below) were analyzed using a StepOnePlus Real-Time PCR System (Applied Biosystems, Waltham, MA, USA) in 96-well plates and the Taqman OpenArray (Applied Biosystems, Waltham, MA, USA) was used as a master mix, as described. SimpleSeq DNA sequencing (Quintara Biosciences, Cambridge, MA, USA) was used to validate PCR products.

### SA-β-gal, EdU, and TEMPOL staining

SA-β-gal staining was performed using the Senescence Detection Kit (Abcam, Cambridge, UK, Cat. No. ab65351) according to the manufacturer’s instructions. Briefly, human aortic endothelial cells (HAECs) were treated with either MGO, DOXO, or MGH-1. For TEMPOL experiments, HAECs were co-treated with 100 μM TEMPOL alongside 250 μM MGO at every media change for seven days, with the media refreshed every alternate day. Control cells were grown in parallel to the same final density as treated cells.

Following the 48-hour incubation period with DOXO/MGH-1 (or seven-day incubation with MGO/TEMPOL), the treatment media was removed, and cells were incubated in standard media for 72 hours. Afterward, cells were treated with 50 μM Gly-Low cocktail for 48 hours. HAECs were then washed, fixed with 4% paraformaldehyde (PFA) for 15 minutes at room temperature, and incubated with the X-gal solution (20 mg/mL) overnight at 37° C in a CO_2_ incubator. Plates containing cells were sealed with parafilm to prevent evaporation and crystal formation. After 16–18 hours, cells were washed twice with PBS.

Following SA-β-gal staining, cells were permeabilized with 0.5% Triton X-100 for 15 minutes at room temperature and stained for EdU using the Click-iT™ EdU Alexa Fluor™ 488 HCS Assay Kit (Invitrogen, Waltham, MA, C10350). For EdU labeling, cells were incubated in medium containing 2.5 mM EdU for 24 hours. After staining, cells were washed once with PBS and incubated with the Click-iT reaction cocktail, prepared according to the manufacturer’s instructions, for 30 minutes in the dark at room temperature. Finally, cells were counterstained with 0.5 mg/mL DAPI for 15 minutes at room temperature, washed with water, and stored in PBS.

SA-β-gal images were captured using bright-field microscopy at 20X magnification and quantified using ImageJ, as previously described [16]. EdU-stained cells were imaged following the FAST protocol [17] using Image Analyst MKII (Image Analyst Software, Novato, CA, USA). Analysis was performed using pipelines described by the FAST protocol. For quantification, SA-β-gal positive cells were normalized to the total number of cells observed in each condition.

**Table:** Table of primer sequences for supplemental data.

**Gene**	**Species**	**Forward primer**	**Reverse primer**
*Cdkn2a*	Mouse	CCCAACGCCCCGAACT	GCAGAAGAGCTGCTACGTGAA
*Cdkn1a*	Mouse	TTGCCAGCAGAATAAAAGGTG	TTTGCTCCTGTGCGGAAC
*Serpine1*	Mouse	TGGAAGGGCAACATGACCAG	TCAGGCATGCCCAACTTCTC
*Lmnb1*	Mouse	GAGCCCCAAGAGCATCCAAT	CTGAGAAGGCTCTGCACTGT
*Gapdh*	Mouse	AAGGTCATCCCAGAGCTGAA	CTGCTTCACCACCTTCTTGA

### Nitric oxide production with MGO/MGH-1 and Gly-low administration

Human aortic endothelial cells (HAECs; PromoCell; donor: female, 80 years old, non-smoker, no known cardiac diseases) were plated in 96-well black-wall glass bottom culture plates (CellVis) and incubated under standard conditions (37° C, 5% CO2) in EGM-2 media supplemented with 50uM/200uM MGO or 200nM MGH-1 in presence of 25uM (for MGH-1/MGO), 50uM (MGO only), or 100uM (MGO only) of Gly-low for 48 hours. All biological samples were tested using 3 experimental replicates. Cells were stained with fluorescent probes to detect NO production (10μM DAR-4M AM; Enzo Life Sciences) before and 6 minutes after addition of 100μM acetylcholine (Sigma-Aldrich, Louis, MO), as an *in vitro* model of endothelial function [18]. Specifically, cells were incubated with DAR-4M AM in HBSS for 45 minutes, after which the dye was washed away with HBSS and imaging was performed after an additional 30-minute incubation at 37° C. DAR-4M AM is a NO specific dye which detects peak NO production 6 minutes after addition of 100μM acetylcholine. This concentration of acetylcholine was chosen because it elicits maximal NO production in cultured HAECs [18]. Hoechst 33342 (Thermo Fisher Scientific) was used as a nuclear stain. Images were acquired using an EVOS M7000 fluorescence microscope (Thermo Fisher Scientific). Fluorescence quantification (difference in NO fluorescence intensity after vs. before the addition of ACh) was performed using Celleste 5.0 Image Analysis Software (Thermo Fisher Scientific).

### Aortic RNA sequencing

RNA was isolated using Zymo research quick RNA miniprep kit (cat # 11-328) according to the manufacturer’s recommendations. Isolated RNA was sent for library preparation and sequencing by Novogene Corporation Inc. where RNA was poly-A selected using poly-T oligo-attached magnetic beads, fragmented, reverse transcribed using random hexamer primers followed by second strand cDNA synthesis using dTTP for non-directional library preparation. Samples underwent end repair, A-tailing, adapter ligated, size selected, amplified, and purified. Illumina libraries were quantified using Qubit and qPCR and analyzed for size distribution using a bioanalyzer. Libraries were pooled and sequenced on an Illumina NovaSeq 6000 to acquire paired-end 150 bp reads. Data quality was assessed, and adaptor reads, and low-quality reads were removed. Reads that passed the quality filtering process were mapped paired end to the reference genome (GRCm38) using Hisat2 v2.0.5. feature Counts v1.5.0-p3 was used to count reads that mapped to each gene. Differential expression analysis was performed using DESEq2 (1.20.0). Where indicated, bootstrapping was performed using R (R version 4.1.2) program ‘boot’ (1.3-28.1). To determine the expected mean and standard deviation, n=i log2 fold changes were randomly selected 1000 times, in which i is the number of genes in the gene set.

### Quantification of methylglyoxal

200 μL of 80:20 MeOH:ddH_2_O (−80° C) containing 50 pmol ^13^C_3_-MGO was added to 10 μL of serum and extracted at −80° C overnight. Insoluble protein was removed via centrifugation at 14,000 x g for 10 min at 4° C. Supernatants were derivatized with 10 μL of 10 mM o-phenylenediamine for 2 h with end-over-end rotation protected from light. Derivatized samples were centrifuged at 14,000 x *g* for 10 min, and the supernatant was chromatographed using a Shimadzu LC system equipped with a 150 x 2mm, 3μm particle diameter Luna C_18_ column (Phenomenex, Torrance, CA) at a flow rate of 0.450 mL/min. Buffer A (0.1% formic acid in H_2_O) was held at 90% for 0.25 min, then a linear gradient to 98% solvent B (0.1% formic acid in acetonitrile) was applied over 4 min. The column was held at 98% B for 1.5 min, washed at 90% A for 0.5 min, and equilibrated to 99% A for 2 min. Multiple reaction monitoring (MRM) was conducted in positive ion mode using an AB SCIEX 4500 QTRAP with the following transitions: *m/z*145.1→77.1 (MGO); *m/z* 235.0→157.0 (^13^C_3_-MGO, internal standard).

### Quantification of MGH-1

10 μL of serum was added to 200 μL of 80:20 MeOH:ddH_2_O (−80° C) containing ten pmol ^13^C-MG-H1 and extracted at −80° C overnight. Insoluble protein was removed via centrifugation at 14,000 x g for 10 min at 4° C, and supernatants were transferred to a new tube. 15 μL of heptafluorobutyric acid (1:1 in H_2_O) was added to each sample, and debris was removed via centrifugation at 14,000 × *g* for 10 min. Samples were analyzed as described previously [13]. (QuARKMod).

### Statistical analyses

Statistical analyses were performed using GraphPad Prism version 10 (GraphPad Software, Inc., San Diego, CA, USA; RRID:SCR_002798). Data were assessed for statistical outliers using the ROUT test (Q = 1%), and identified outliers were excluded from final analyses. Differences in pulse wave velocity (PWV) in the young male mouse study, and NO production in response to MGO/MGH-1 exposure were analyzed using one-way analysis of variance (ANOVA), followed by Tukey’s multiple comparison test when a main effect was detected. Senescence marker expressions following Doxo/MGO/MGH-1 treatment were assessed using two-way ANOVA with Sidak’s multiple comparisons test applied when a main effect was observed. For the young female PWV study, old male and female PWV study, plasma MGO, and MGH-1 levels (in young and old cohorts), comparisons were made using unpaired t-tests. *Ex vivo* superoxide levels, elastic modulus, and immunofluorescence data were analyzed using paired t-tests, as different media conditions were tested on aortas obtained from the same mouse. All data are presented as mean ± SEM, and statistical significance was set at P < 0.05.

## RESULTS

### Chronic MGO-induced glycation stress induces aortic stiffening in young mice

**Figure 1:**
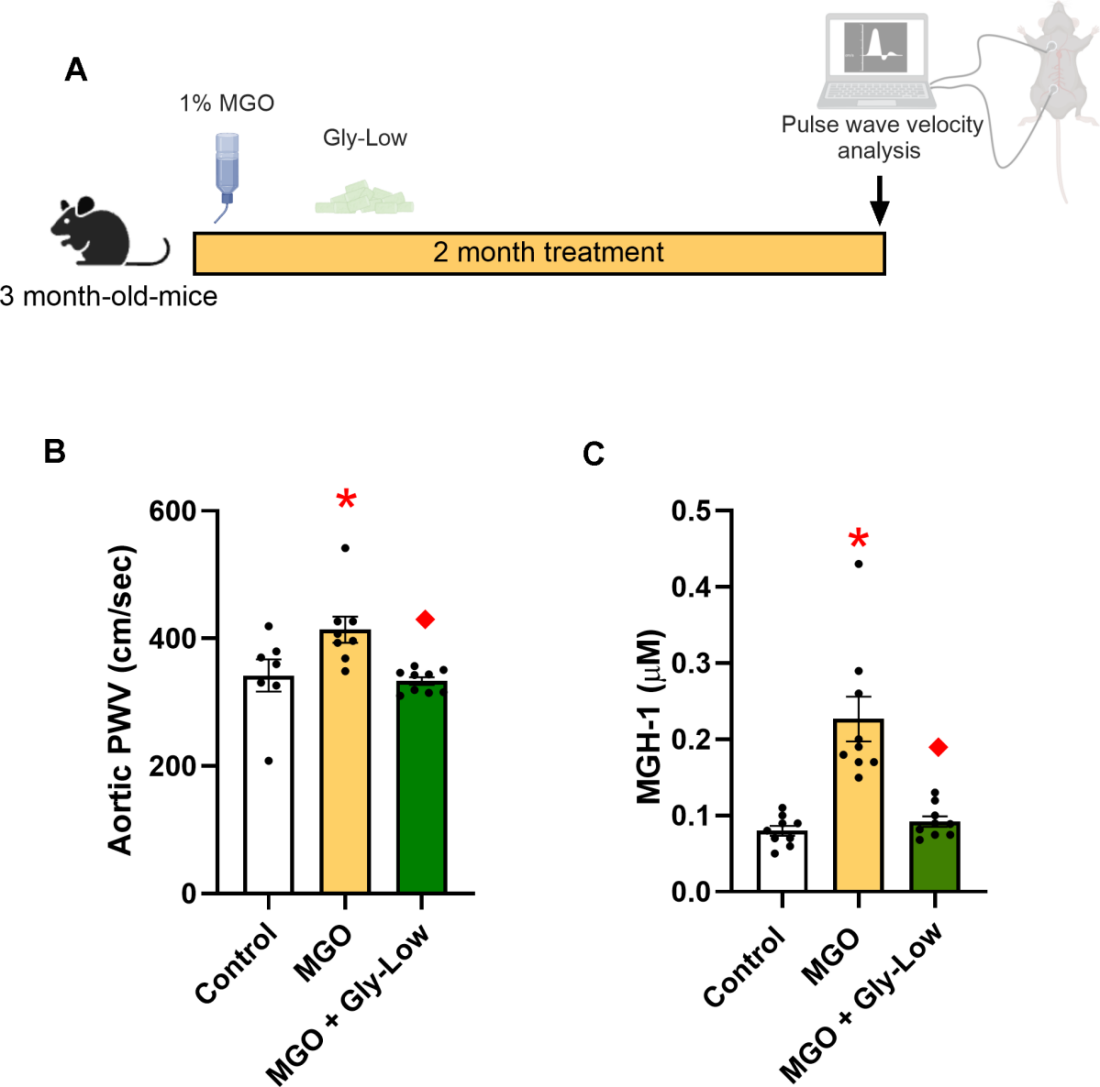
**Methylglyoxal (MGO) induces glycation stress which can be prevented by glycation lowering (Gly-Low) compound supplementation.** (**A**) Treatment paradigm for MGO and Gly-Low supplementation in 3-month-old male mice. (**B**) Aortic PWV measured after intervention (n=7–10/group). (**C**) Plasma MGH-1 levels in male mice that received normal/Gly-Low chow with MGO supplemented in water (n=9/group). One-way ANOVA was used for panel (**B**), and an unpaired t-test was performed for panel (**C**). All values are in mean ± SEM, *p<0.05 vs. control; ♦p<0.05 vs. MGO alone.

As a first step in elucidating the impact of MGO-induced glycation stress, we established a glycation stress model through the pharmacological administration of MGO in drinking water at a 1% concentration in young adult male mice and compared them to young adult mice that received regular drinking water without MGO, as previously described [19]. This model allows for the simulation of chronic glycation stress in a controlled environment independent of aging. Following two months of daily MGO consumption, aortic PWV was measured *in vivo* to evaluate MGO-induced aortic stiffening (Figure 1A). Aortic PWV is the gold-standard non-invasive approach for assessing aortic stiffness and is corollary to the reference standard measure of aortic stiffness in humans, carotid-femoral PWV [20]. Chronic MGO consumption was associated with an ~20% higher aortic PWV in young adult male mice (MGO, 414 ± 21 vs. control, 342 ± 25 cm/sec; *P*=0.03) (Figure 1B). Additionally, MGO consumption caused a 2-fold elevation in circulating MGH-1 (*P*<0.001 vs. control) (Figure 1C) and previous research has shown that Gly-Low supplementation significantly lowers circulating MGH-1 levels in young mice [13]. To determine if glycation stress-induced aortic stiffening can be prevented by inhibiting MGO, an additional group of mice were treated with glycation lowering compounds (Gly-Low) concurrently with MGO. We found that Gly-Low effectively prevented the MGO-induced increase in aortic PWV (MGO + Gly-low, 333 ± 6 cm/sec; *P* = 0.009 vs. MGO; *P*=0.93 vs. Control) (Figure 1B). Similar results were observed in young adult female mice, such that MGO-supplemented mice had higher aortic PWV relative to animals that consumed the control chow (Control, 376 ± 29 vs. MGO, 472 ± 23 cm/sec; *P*=0.02) (Supplementary Figure 1A).

These findings emphasize the direct pathogenic role of MGO-induced glycation stress in aortic stiffening and illustrate the therapeutic potential of glycation-lowering compounds, like Gly-Low, in preventing aortic stiffening associated with glycation stress.

### Direct effect of MGO and Gly-Low on the *ex-vivo* stiffening on aorta rings

**Figure 2:**
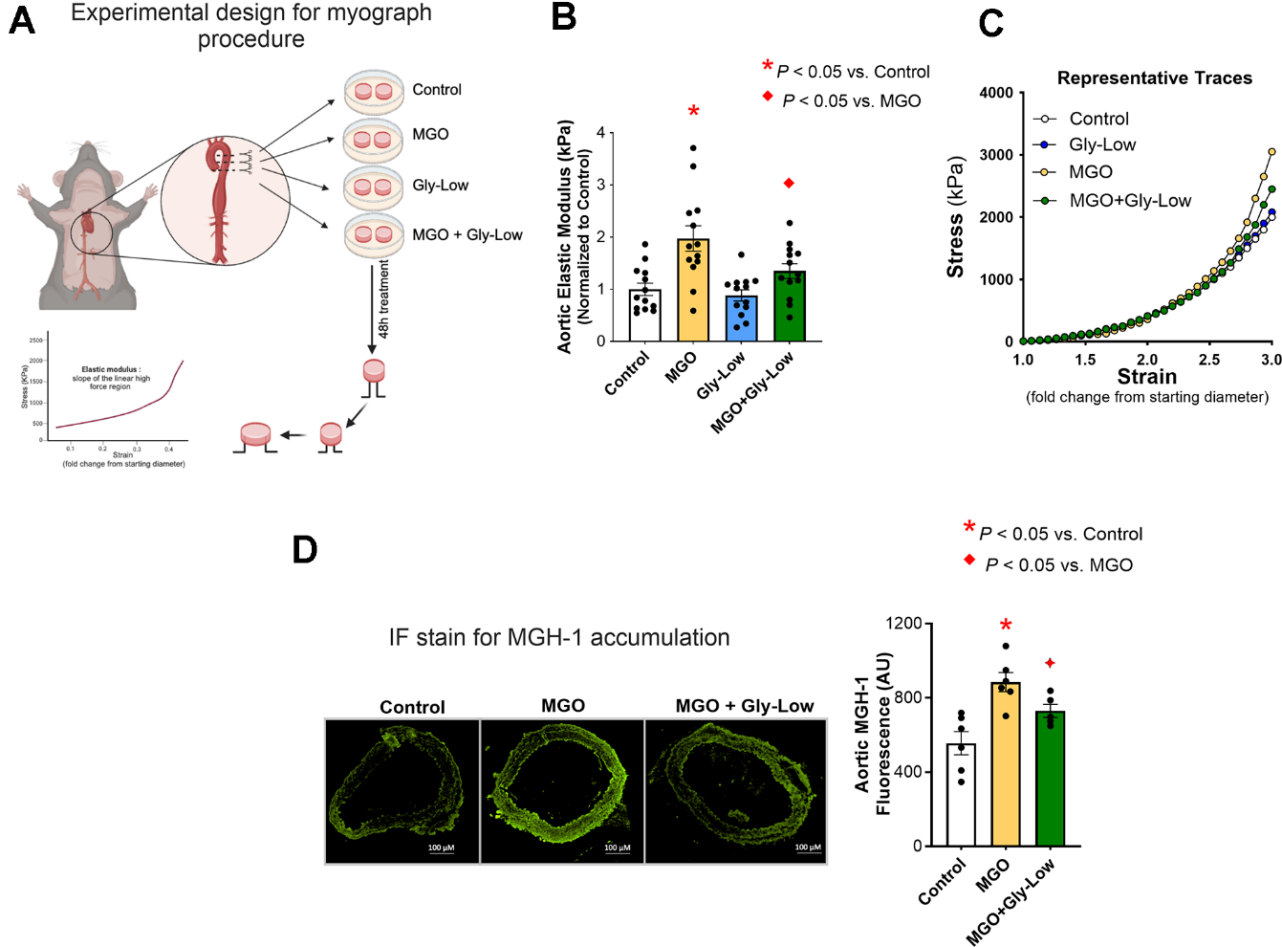
***Ex-vivo* incubation with MGO increases arterial stiffness which is mitigated by Gly-Low coincubation as measured by aortic elastic modulus.** (**A**) Experimental design for the myograph procedure. (**B**) Aortic elastic modulus for young (6-month) intervention-naïve male mouse aortic rings following exposure to standard media (control), MGO, Gly-Low, and MGO+Gly-Low (n=13/group). (**C**) Representative curves for wire myography experiment. (**D**) Representative immunofluorescence staining for methylglyoxal-derived hydroimidazolone-1 (MGH-1) accumulation in aortic rings exposed to standard media (control), MGO, and MGO+Gly-Low (n=5–6/group). Panels (B) and (D) were analyzed using paired t-tests, as different media conditions were tested on aortas obtained from the same mouse. All values are in mean ± SEM, *p<0.05 vs. control; ♦p<0.05 vs. MGO alone.

Next, to investigate the potential impact of MGO and Gly-Low on the intrinsic material stiffness of the aortic wall, we conducted *ex vivo* experiments using excised aorta rings from intervention-naive young adult (6 months old) male C57BL/6 mice and performed assessments of elastic modulus. Excised aorta rings from intervention-naïve young adult mice were incubated for 48 hours under the following conditions: (*1*) DMEM + 1% penicillin-streptomycin (control standard media), (*2*) Standard media + 500 μM MGO, (*3*) Standard media + 100 μM Gly-Low, and (*4*) Standard media + 500 μM MGO + 100 μM Gly-Low. Aortic elastic modulus assessments were subsequently performed as described previously [15] (Figure 2A). Aortic rings treated with MGO exhibited a 2-fold increase in elastic modulus (*P*=0.003 vs. control condition), indicative of increased intrinsic aortic wall stiffness (Figure 2B). This MGO-induced aortic wall stiffening was attenuated by co-treatment with Gly-Low (*P=*0.009 MGO vs. MGO + Gly-Low) (Figure 2B). Notably, Gly-Low treatment alone had no off-target effects on aortic elastic modulus (i.e., no difference between control and Gly-Low alone, *P*=0.40), confirming its specificity to MGO-induced glycation stress (Figure 2B). No changes were observed in the aortic diameter and wall thickness after incubation of aortas in the aforementioned conditions (Supplementary Table 1). MGO exposure increased the accumulation of the MGO-derived AGE, MGH-1 (*P*=0.004, MGO vs. Control) (Figure 2C), and co-exposure of MGO with Gly-Low markedly attenuated this increase in MGH-1 expression (P=0.04, MGO vs. MGO+Gly-Low). The latter demonstrates molecular target engagement and further supporting its protective role against MGO-induced glycation stress (Figure 2C). Together, these findings demonstrate that MGO-induced glycation stress directly increases aortic stiffness and promotes AGE accumulation in arteries, independent of changes in aortic diameter and wall thickness.

### MGO-induced excess oxidative stress drives aortic stiffening

**Figure 3:**
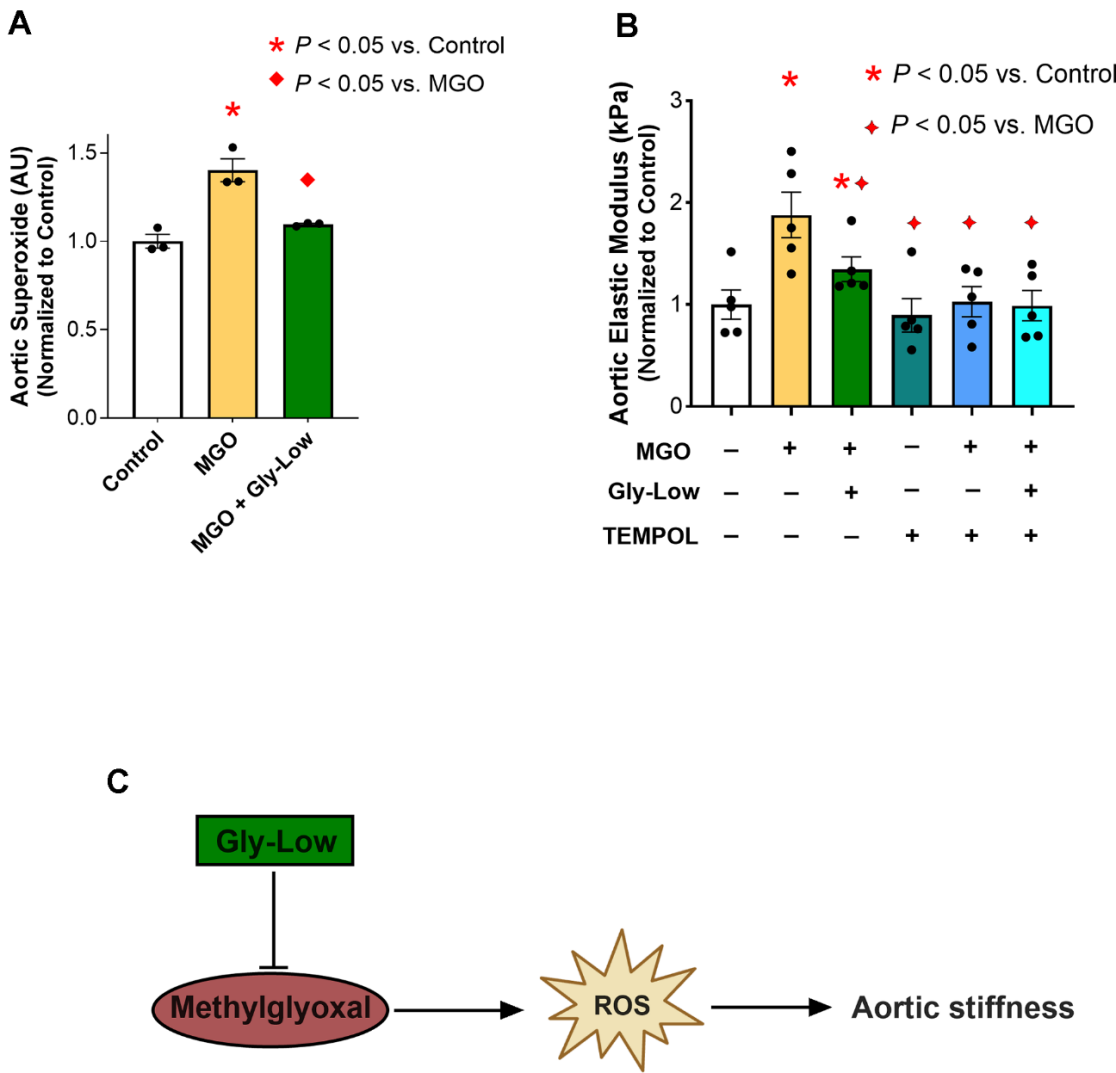
**MGO-induced glycation stress drives aortic stiffness, in part, by increasing reactive oxygen species (ROS) levels.** (**A**) Superoxide levels assessed via Electron Paramagnetic Resonance spectroscopy in young intervention-naïve male mouse aortic rings following exposure to standard media (control), MGO, and MGO+Gly-Low (n=3/group). (**B**) Elastic modulus in aortic rings incubated with/without superoxide scavenger TEMPOL (n=5/group). (**C**) Mechanistic schematic depicting how MGO-induced glycation stress causes aortic stiffness via increasing ROS levels. Panels (**A**, **B**) were analyzed using paired t-tests, as different media conditions were tested on aortas obtained from the same mouse. All values are in mean ± SEM, *p<0.05 vs. control; ♦p<0.05 vs. MGO alone.

Elevated glycation stress has been shown to induce excessive reactive oxygen species (ROS)-related oxidative stress bioactivity in various cell types [14, 21]. Considering the established role of excessive ROS bioactivity in aortic stiffening [7], we next sought to investigate whether MGO-induced aortic stiffening is mediated through ROS-dependent mechanisms. To accomplish this, we first assessed superoxide bioactivity in aorta rings from intervention-naïve male mice via electron paramagnetic resonance spectroscopy (reference standard experimental approach for assessing ROS in biological tissues) [21, 22]. Excised aorta rings were incubated for 48 hours under the following conditions: (*1*) Standard media (Control), (*2*) Standard media + 500 μM MGO, and (*3*) Standard media + 500 μM MGO + 100 μM Gly-Low. Incubation with MGO alone led to an ~40% increase in aortic superoxide levels (*P*=0.04 vs. Control), and concomitant incubation of Gly-Low with MGO prevented this increase in superoxide bioactivity (*P*=0.03 MGO vs. MGO+GlyLow) (Figure 3A).

Next, to interrogate the direct role of excessive ROS bioactivity in aortic stiffening induced by MGO-mediated glycation stress, we incubated a new cohort of aorta rings from young adult intervention naïve mice for 48 hours with the superoxide scavenger, TEMPOL (1 μM), in the presence of MGO and Gly-Low, and assessed elastic modulus as described above. As previously demonstrated, incubation with MGO alone again caused a 75% increase in elastic modulus compared to control condition (*P*=0.002), indicating MGO-induced aortic stiffening, which was fully prevented by coincubation with TEMPOL (*P*=0.01). Additionally, when aortic rings were incubated with MGO and Gly-Low, the increase in aortic stiffening was attenuated (corroborating our findings presented in Fig 2) (Figure 3B). Moreover, the combined incubation with MGO, TEMPOL, and Gly-Low completely prevented the increase in stiffness, suggesting Gly-Low likely prevented MGO-mediated aortic stiffening by mitigating excessive ROS bioactivity (Figure 3B). Notably, TEMPOL alone did not produce any off-target effects on elastic modulus, as evidenced by the minimal effects on the control condition (Figure 3B). These data suggest that MGO-induced glycation stress contributes to aortic stiffening, in part, through excessive ROS bioactivity (Figure 3C), underscoring the potential of targeting glycation stress as a therapeutic strategy for mitigating excessive ROS-mediated aortic stiffening.

### MGH-1 directly induces aortic stiffening

**Figure 4:**
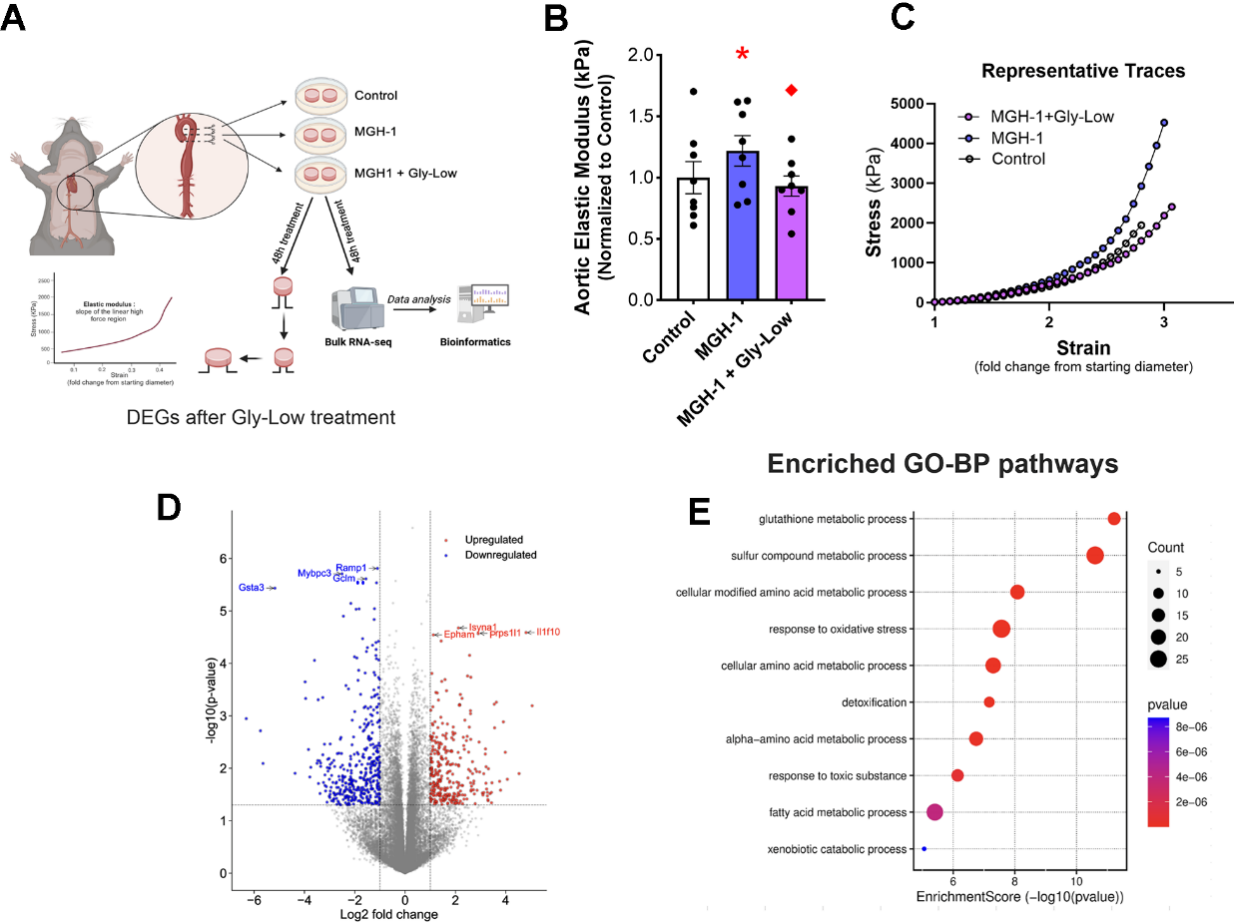
**MGH-1 can increase aortic stiffness in young mouse aortas and gly-Low supplementation prevents it.** (**A**) Study paradigm for the *ex-vivo* aortic stiffness measurement after 48-hour incubation with standard media (control), MGH-1, and MGH-1+Gly-Low. (**B**) Aortic elastic modulus for young intervention-naïve male mouse aortic rings after incubation (n=8/group). (**C**) Volcano plot showing the differential expressed genes (510 DEGs) with Padj < 0.05. (**D**) Gene ontology analysis of significantly upregulated (280) differentially expressed genes in response to Gly-Low treatment showing enriched biological processes. Panel (**B**) was analyzed using paired t-tests, as different media conditions were tested on aortas obtained from the same mouse. All values are in mean ± SEM, *p<0.05 vs. control; ♦p<0.05 vs. MGH-1 alone.

MGH-1 is a prevalent AGE associated with various age-related disorders [12, 23]. MGH-1, a non-crosslinking AGE, is predominantly produced from MGO and has been implicated in CVD [12, 24], but its direct effect on aortic stiffening is unknown. Thus, we aimed to determine if MGH-1 directly influences intrinsic mechanical wall stiffness of the aorta (elastic modulus) and whether Gly-Low could prevent this effect. To investigate this, aorta rings were excised from young intervention naïve mice for 48 hours under the following conditions: (*1*) Standard media (control), (*2*) Standard media + 200 nM MGH-1, and (*3*) Standard media + 200 nM MGH-1 + 100 μM Gly-Low. Aortic elastic modulus assessments were subsequently performed as described above. Additionally, RNA sequencing was conducted on aortic segments incubated with MGH-1 and MGH-1 + Gly-Low to determine the molecular events in arteries altered by glycation stress and how these processes are influenced by a glycation stress lowering compound (Figure 4A).

Following the 48-hour incubation period, incubation with MGH-1 increased (*P*=0.04 vs. control) aortic elastic modulus, an effect that was blocked by Gly-Low coincubation (*P*=0.02, MGH-1 vs. MGH-1+GlyLow) (Figure 4B). To further dissect the molecular changes induced by Gly-Low, we performed bulk RNA sequencing on Gly-Low–treated aortic rings in the presence and absence of the MGH-1. Differentially expressed genes (DEGs) were defined as those with adjusted p-value (Padj) < 0.05 and fold-change ≥ 1.5. In total, 510 DEGs were identified—displayed as a volcano plot (Figure 4C)-including 280 upregulated and 230 downregulated genes by Gly-Low treatment.

Gene Ontology Biological Process enrichment was carried out exclusively on these 280 upregulated DEGs, revealing detoxification pathways and the “glutathione metabolic process” among the top hits (Figure 4D). Finally, MCODE clustering of the protein–protein interaction network built from upregulated DEGs delineated five major modules-glutathione biosynthesis and oxidative-stress response (Supplementary Figure 2). Together, these data indicate that Gly-Low elicits detoxification pathways, in line with the reduced ROS bioactivity observed in our preceding *ex vivo* experiments.

### MGO induces cellular senescence in aortic endothelial cells independent of ROS production

**Figure 5:**
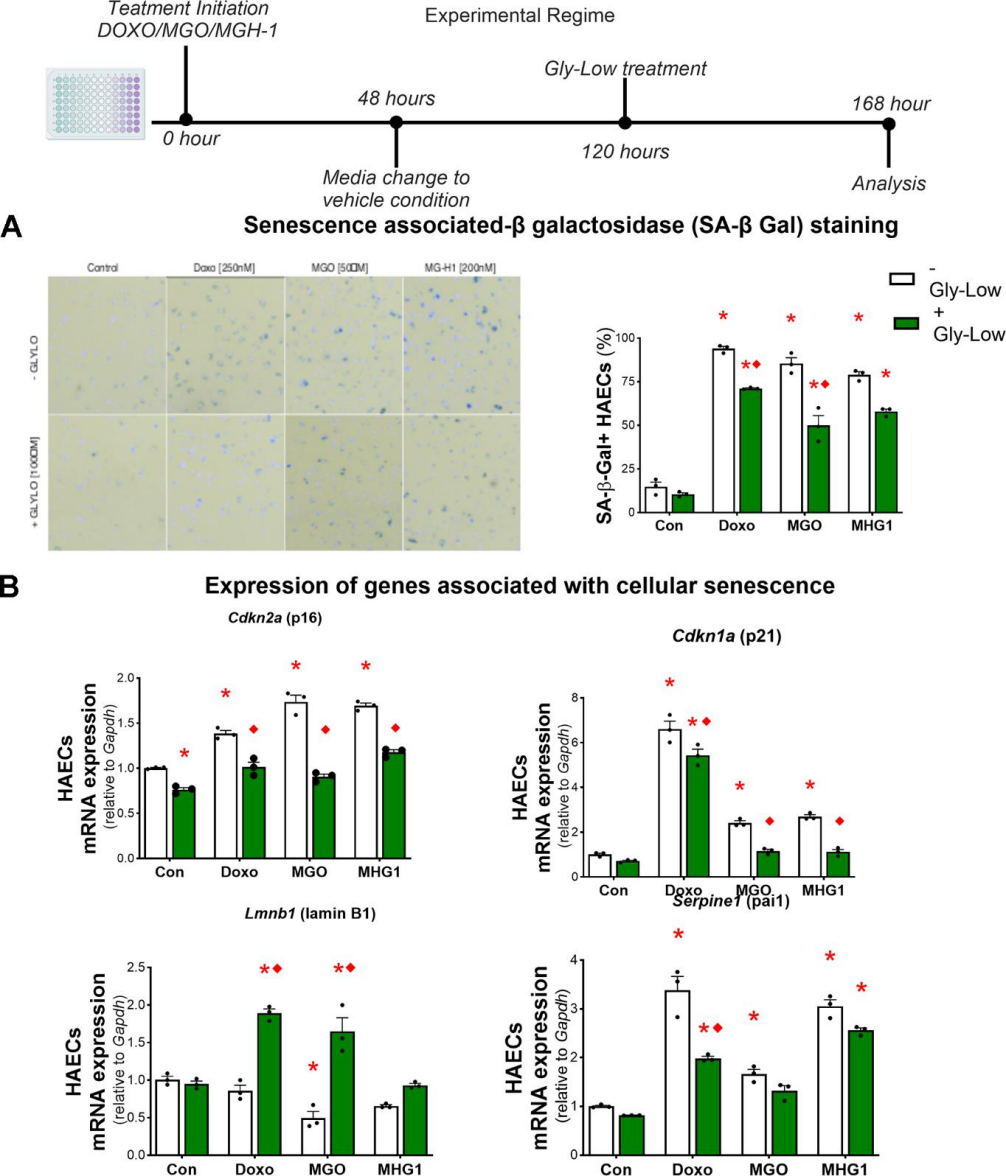
**MGO-induced glycation stress can increase cellular senescence.** (**A**) Senescence-associated β-galactosidase staining for HAECs treated with DOXO/MGO/MGH-1 with or without Gly-Low supplementation. (**B**) mRNA expression of various senescence-associated genes. All data were analyzed using two-way ANOVA, as the analysis included both the treatment condition (control, DOXO, MGO, MGH-1) and the second factor (with or without Gly-Low). All values are in mean ± SEM, *p<0.05 vs. control (–Gly-Low); ♦p<0.05 vs. (–Gly-Low) of the same condition.

Endothelial cell senescence directly promotes aortic stiffening and vascular oxidative stress [15, 25]. Given that MGO-mediated glycation stress induced aortic stiffening by increasing ROS bioactivity in the present study, and that cellular senescence has been implicated as a cause of excessive ROS production in endothelial cells and intact arteries [26, 27], we next hypothesized that MGO-induced glycation stress and MGH-1 may cause cellular senescence in endothelial cells. To test this hypothesis, we treated HAECs with MGO, MGH-1, and DOXO (which served as a positive control for senescence induction [28]) (Figure 5A). We observed that MGO and MGH-1 caused an increase in senescence biomarkers similar to DOXO, as evidenced by greater SA-β-gal intensity (Figure 5B) and higher expression of canonical senescence genes such as *Cdkn1a* (p21), *Cdkn2a* (p16) and *Serpine1* (PAI-1), and lower *Lmnb1* (Lambin B1) (Figure 5B). In all conditions, lowering glycation stress via Gly-Low exposure generally mitigated the induction of cellular senescence (Figure 5A, 5B). These findings indicate that MGO-induced glycation stress is a driver of cellular senescence in endothelial cells.

To determine whether these senescence-promoting effects were mediated through excessive ROS production, we treated HAECs with MGO in the presence and absence of the ROS scavenger TEMPOL and subsequently assessed canonical senescence markers. Our results demonstrated that TEMPOL treatment had no effect on MGO-induced cellular senescence in HAECs (Supplementary Figure 3A–3C), implying that MGO-induced cellular senescence in endothelial cells likely occurs through mechanisms that are independent of excessive ROS bioactivity and that cellular senescence likely precedes ROS production as a result of MGO-induced glycation stress.

### MGO lowers NO production in aortic endothelial cells

We have previously demonstrated that a key characteristic of senescent endothelial cells is reduced NO production [15]. Thus, to further characterize the influence of glycation stress on characteristics of endothelial cell senescence, we sought to determine whether glycation stress lowered NO production in endothelial cells. To accomplish this, HAECs were incubated with standard media, 50 μM MGO, or 200 μM MGO. MGO concentrations significantly decreased NO production compared to control (50 μM: *P* = 0.02; 200 μM: *P* = 0.0008) (Supplementary Figure 4A). We then hypothesized that co-incubation with Gly-Low could mitigate this reduction. Indeed, Gly-Low at all tested concentrations (20 μM, 50 μM, 100 μM) significantly prevented the MGO-induced decline in NO production (MGO vs. 25 μM: *P* = 0.001; MGO vs. 50 μM: *P* = 0.005; MGO vs. 100 μM: *P* = 0.001) (Supplementary Figure 4B). Next, we examined whether MGH-1 similarly affects NO production. Incubation with MGH-1 also led to a reduction in NO production (~20%; *P* = 0.09), while co-incubation with Gly-Low preserved NO levels (*P* = 0.04, MGH-1 vs. MGH-1 + Gly-Low) (Supplementary Figure 4C). Together, these findings are consistent with our previous observations of lower NO production in senescent endothelial cells [15] and further confirm the senescence-inducing effects of MGO-induced glycation stress, and protective effects of Gly-Low on endothelial cell senescence-related phenotypes.

### Gly-Low mitigates senescence-induced aortic stiffening by activating glyoxalase-1 mediated detoxification pathway

**Figure 6:**
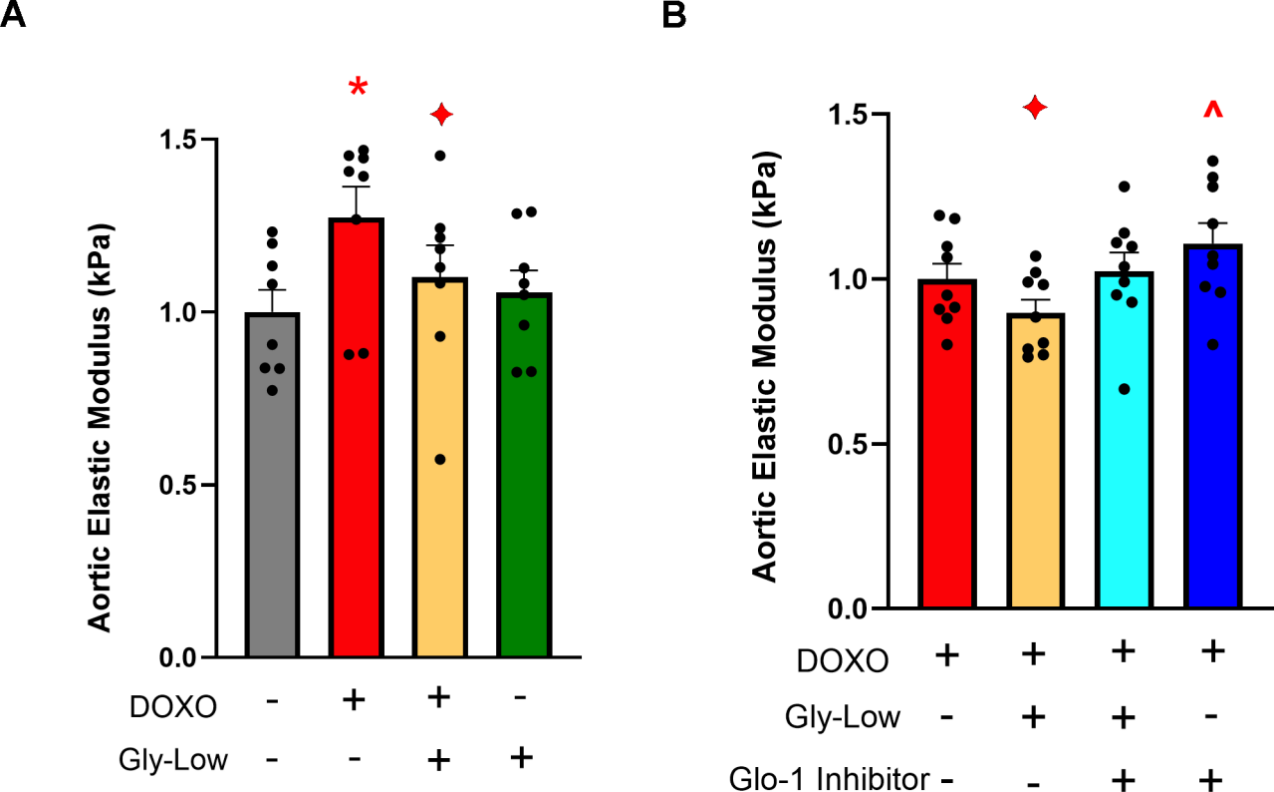
**Gly-Low supplementation mitigates DOXO-induced aortic stiffening.** (**A**) Aortic elastic modulus in young intervention-naïve male mouse aortas after 48-hour incubation with DOXO/Gly-Low (n=8/group). (**B**) Aortic elastic modulus in young intervention-naïve male mouse aortas after 48-hour incubation with DOXO/Gly-Low in the absence/presence of a Glo-1 inhibitor (n=9/group). All data were analyzed using paired T-test, as different media conditions were tested on aortas obtained from the same mouse. All values are in mean ± SEM, *p<0.05 vs. control (No DOXO/No Gly-Low); ♦p<0.05 vs. DOXO alone; ^p<0.05 vs. DOXO+Gly-Low.

Preliminary studies from our laboratory have demonstrated that DOXO promotes aortic stiffening as a result of excessive cellular senescence [29]. Moreover, our RNA sequencing analysis indicated that Gly-Low may exert its anti-glycation stress properties by activating detoxification pathways. Thus, we next hypothesized that Gly-Low may mitigate the effects of senescence-induced (via DOXO exposure) aortic stiffening by activating the detoxification system. To test this hypothesis, we incubated excised aorta rings from young intervention naïve male and mice for 48 hours under the following conditions: (*1*) Standard media (control), (*2*) Standard media + 1μM DOXO, (*3*) Standard media + 1 μM DOXO + 100 μM Gly-Low, and (4) Standard media + 100 μM Gly-Low. DOXO-incubated aorta rings exhibited higher aortic stiffness compared to the control condition (*P*=0.006). However, concomitant incubation of DOXO and Gly-Low prevented the increase in aortic stiffening observed with DOXO (*P*=0.02; DOXO + Gly-Low vs. DOXO alone) (Figure 6A), indicating that cellular senescence is likely a mechanism underlying glycation stress-induced aortic stiffening.

Considering the canonical role of the Glyoxalase-1 (Glo-1) detoxification system in clearing excessive MGO-induced glycation stress [30], we next investigated whether the effects of Gly-Low were mediated through activation of the Glo-1 signaling pathway. Aortic rings incubated with an established Glo-1 inhibitor [31] in the presence of DOXO and Gly-Low showed no change in stiffness compared to the DOXO group alone (*P*=0.15). Additionally, aorta rings incubated with DOXO in the presence of the Glo-1 inhibitor exhibited significantly increased (*P*=0.03) aortic stiffness compared to DOXO alone (Figure 6B). Collectively, these results suggest that DOXO-mediated aortic stiffening is induced by glycation stress, in part via suppression of Glo-1, and Gly-low mechanistically exerts its effect through activation of the Glo-1 pathway. Furthermore, our RNA sequencing data validates that Gly-Low mediates its therapeutic effect in part by activating the Glo-1 detoxification pathway. This suggests that activating or preserving Glo-1 may be a potential strategy for mitigating excessive cellular senescence-induced aortic stiffening.

### Reducing glycation stress may improve age-associated aortic stiffening

**Figure 7:**
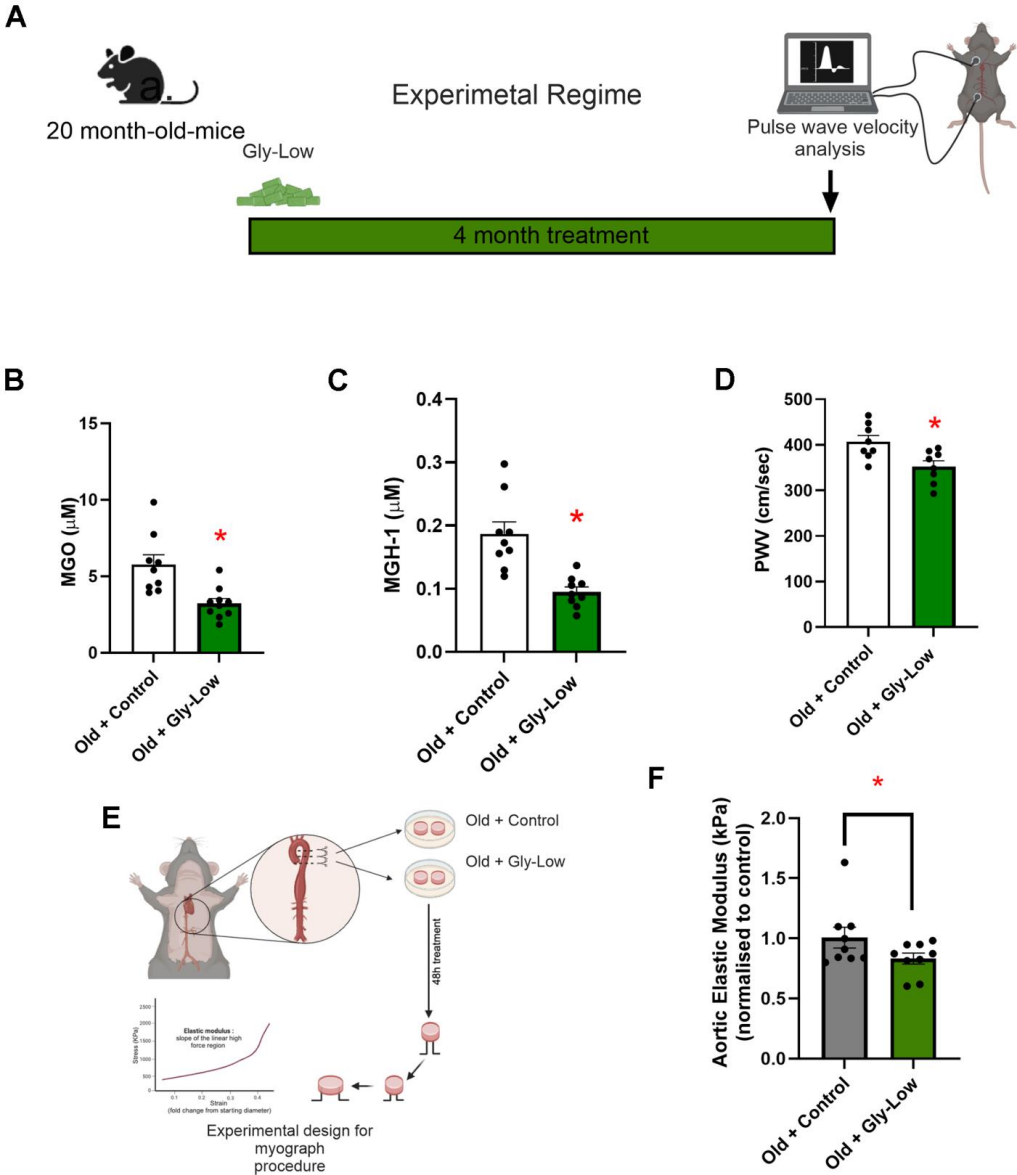
**Gly-Low lowers MGO-induced glycation stress and aortic stiffening in old mice.** (**A**) Treatment paradigm for Gly-Low supplementation in 20-month-old male mice. (**B**) Aortic stiffness as measured by PWV between two study groups (n=8/group). (**C**) MGO levels in plasma of mice that received control or Gly-Low enriched diet (n=9–10/group). (**D**) Plasma MGH-1 levels (n=9/group). (**E**, **F**) *Ex-vivo* study paradigm focusing on aortic stiffness in intervention-naïve male old (24–26 months) mouse aortas after 48-hour incubation in standard media (control) and Gly-Low (n=8). Panels (**B**), (**C**), and (**D**) were analyzed using unpaired t-tests, while panel (**F**) was analyzed using a paired t-test, as different media conditions were tested on aortas obtained from the same mouse. All values are in mean ± SEM, *p<0.05 vs. old control.

Building on our findings that MGO-induced glycation stress causally drives aortic stiffening independent of advanced age and given that glycation stress naturally accumulates with aging due to impaired activation of detoxification pathways (e.g., Glo-1) [6], we next sought to determine whether reducing glycation stress through Gly-Low supplementation lowers aortic stiffness in old mice. To test this, we supplemented 20-month-old male mice with Gly-Low in their chow (Figure 7A). After a four-month treatment period, we observed that Gly-Low-supplemented mice had lower aortic PWV than age-matched controls (Old Gly-Low, 352 ± 11 vs. Old control, 407 ± 12 cm/sec; *P*=0.01) (Figure 7B) and similar levels to that of young control mice (342 ± 25 cm/sec Figure 1B). Similar results were also observed in old female mice supplemented with Gly-Low (Old Gly-Low, 395 ± 14 vs. Old control, 456 ± 18 cm/sec, *P*=0.01) (Supplementary Figure 1B). Gly-Low supplementation also lowered circulating concentrations of MGO (*P*=0.002) and the MGO-derived AGE; MGH-1 (*P*=0.01), by ~50% compared to age-matched control mice (Figure 7C, 7D).

To further investigate the direct aortic de-stiffening effects of Gly-Low in the setting of advanced age, we incubated aortic rings obtained from 24–26-month-old mice for 48 hours in the absence and presence of Gly-Low. Following the incubation period, we assessed elastic modulus as described above (Figure 7E). Our results indicated a significant reduction in stiffness in old mouse aorta rings incubated with Gly-Low (*P*=0.04), suggesting a direct role of Gly-Low in lowering aortic stiffness with aging (Figure 7F). Collectively, these findings suggest that excessive glycation stress associated with aging directly contributes to age-related aortic stiffening.

## DISCUSSION

In this study, we investigated the impact of MGO-induced glycation stress on aortic stiffness in young and old mice, explored the potential molecular mechanisms involved, and evaluated the therapeutic potential of the glycation-lowering compound Gly-Low. Our findings provide novel and significant insights into the role of MGO-induced glycation stress as a mediator of age-related aortic stiffening and offer proof-of-principle efficacy for using glycation-lowering compounds to mitigate this process. While MGO has previously been implicated in endothelial dysfunction and vascular lacking. Our results demonstrate that chronic MGO exposure significantly increases aortic stiffness in young mice. This effect was particularly pronounced in our pharmacological model of glycation stress, where young adult mice exhibited a marked increase in aortic stiffness after just two months of MGO exposure. Lastly, we also demonstrate the direct influence of glycation stress in mediating age-related aortic stiffening, which underscores the critical role of AGEs in promoting aortic stiffening with aging. Notably, our results also reveal the direct impact of MGO on aortic stiffening, supporting the notion that MGO-induced glycation stress can independently drive this pathology.

MGH-1, a predominant AGE derived from MGO, has been shown to act through non-crosslinking, receptor-mediated pathways to influence cellular and physiological function; here, our *ex vivo* experiments demonstrate that MGH-1 directly increases aortic stiffness. Although a recent clinical cross-sectional study found no independent association between fasting MGO levels and large artery stiffness after multivariable adjustment [32], our study directly demonstrates that glycation stress increases aortic stiffness, providing functional evidence beyond observational correlations. These findings suggest that MGO-derived glycation stress drives AGE accumulation and induces functional—rather than purely structural—alterations in the arterial wall to promote aortic stiffening. Previous studies have shown that AGE accumulation contributes to other CV-related complications such as aortic stenosis [3], myocardial stiffening [33], and endothelial dysfunction [23], and findings from the present study extend previous observations by establishing a direct role for MGO in mediating aortic stiffening.

A key mechanism gleaned from the results of the present study is the role of excessive ROS in mediating glycation stress-induced aortic stiffening. Prior work has identified excessive aortic oxidative stress as a hallmark of age-related aortic stiffening [7, 14]. Our study extends these findings by identifying increased glycation stress as an upstream driver of excess aortic ROS bioactivity. MGO exposure to aortic rings from young adult animals led to a substantial increase in superoxide production, an effect significantly attenuated with Gly-Low exposure. The use of the ROS scavenger TEMPOL validated the involvement of oxidative stress in this process, as TEMPOL exposure effectively prevented MGO-induced aortic stiffening. This highlights the critical role of ROS in the pathogenesis of glycation stress-associated aortic stiffening. However, large-scale randomized controlled trials using chronic antioxidant supplementation have failed to demonstrate improvements in arterial health [32], underscoring the need for alternative therapeutic strategies that mitigate excessive oxidative stress. Thus, our findings suggest that targeting glycation stress may be a promising strategy to blunt excess ROS-related aortic stiffening.

Additionally, our results on the induction of cellular senescence by MGO and MGH-1 provide further insight into how glycation stress may promote aortic stiffening. While MGO has been previously shown to induce senescence in the brain [34] our study demonstrates that MGO exposure of aortic endothelial cells can directly induce a senescent phenotype. The lack of effect of TEMPOL on MGO-induced senescence suggests that senescence may drive excess ROS production—rather than simply result from it—under conditions of glycation stress. Finally, we show that both MGO and the MGO-derived AGE, MGH-1, induce endothelial dysfunction (evidenced by reduced NO production), an effect that was prevented with Gly-Low. Previous studies have shown the senescent endothelial cells have lower NO production, relative to non-senescent endothelial cells, which can directly increase aortic stiffness [35]. These findings highlight the complex interplay between glycation stress, senescence, ROS, NO production, and aortic stiffening.

Another significant finding from our study is the involvement of the glyoxalase-1 (Glo-1) detoxification pathway in mediating the therapeutic effects of Gly-Low. The glyoxalase system is a key regulator of endogenous glycation detoxification [30], and Glo-1 activity has been shown to decline with advancing age [35], contributing to increased MGO accumulation [33]. Our RNA sequencing results indicate that Gly-Low activates detoxification pathways, which likely contribute to the reduction of MGO-induced glycation stress. The inhibition of the Glo-1 pathway abolished the protective effects of Gly-Low, confirming that this detoxification system is essential for mitigating glycation stress-induced aortic stiffening.

Several clinical trials targeting AGE accumulation using crosslink breakers such as ALT-711 or scavengers like aminoguanidine have yielded modest or inconclusive outcomes in terms of the efficacy of these compounds in reducing arterial stiffness [33, 36–38]. These limited outcomes raise questions about the clinical utility of such compounds in reversing established large artery stiffening. In addition, aminoguanidine has been associated with adverse effects, including autoantibody production and flu-like symptoms [38], which have limited its clinical utility. In contrast, Gly-Low is composed of naturally derived compounds, potentially offering a more favorable safety profile and improved tolerability for long-term administration.

The therapeutic potential of Gly-Low was supported across our *in vitro*, *ex vivo*, and *in vivo* models. In young mice, Gly-Low significantly mitigated MGO-induced glycation stress, which was accompanied by a marked prevention of aortic stiffening. These findings, along with our *ex vivo* results showing that Gly-Low co-incubation prevents the MGO-induced increase in stiffness in young mouse aortic rings, provide direct evidence that targeting glycation stress can blunt aortic stiffening. In old mice, Gly-Low lowered circulating levels of MGH-1 — an MGO-derived AGE that we show can directly increase aortic stiffness — and this reduction was accompanied by lower aortic stiffness. These results suggest that the elevated glycation stress burden with aging contributes mechanistically to aortic stiffening and that Gly-Low mitigates this process by reducing AGE accumulation. Collectively, our findings support Gly-Low as a promising therapeutic strategy to combat age-related vascular dysfunction driven by glycation stress.

## CONCLUSION

Our study elucidates the putative molecular-cellular mechanisms by which MGO-induced glycation stress contributes to aortic stiffening and highlights the therapeutic potential of Gly-Low in mitigating these effects. By targeting glycation stress, reducing oxidative stress and cellular senescence, and by activating endogenous detoxification pathways, Gly-Low offers a promising strategy for preserving arterial health and potentially preventing the progression of CVD associated with aging or other conditions that may promote glycation stress in arteries (e.g., metabolic disorders [6, 36]). Further research is warranted to explore the clinical applicability of Gly-Low and other glycation-lowering compounds in managing arterial stiffness and improving CV outcomes in at-risk populations.

### Limitations

This study has several limitations that should be considered when interpreting the findings. First, although we examined both young and old mice, they were studied in separate experimental cohorts. Age-related increases in aortic stiffness and elastic modulus have been well documented in C57BL/6J mice [39], but young mice were not included in the aging arm of this study to avoid redundancy. Second, while we monitored food and water intake, subtle changes in consumption cannot be entirely excluded as partial contributors to the observed outcomes. We have noted this possibility, although our *ex vivo* aortic ring experiments and high-fat, high-carbohydrate diet studies in young mice suggest that the vascular benefits of Gly-Low are largely independent of caloric restriction [13]. Third, PWV was measured under isoflurane anesthesia, which can influence heart rate and thus vascular parameters. Although all groups were treated identically, anesthesia remains a potential confounder, and future studies should incorporate direct heart rate monitoring. Fourth, while we used MGO as a representative reactive carbonyl, additional AGE precursors such as glyoxal or 3-deoxyglucosone may also contribute to aortic stiffening. Future work will be necessary to determine whether the observed effects are unique to MGO or reflect a broader carbonyl stress response. Finally, we assessed arterial stiffness and elasticity through PWV and tensile testing, which are well-established surrogates of vascular compliance [40], but direct compliance measurements under physiological loading were not performed. Lastly, although both male and female mice exhibited MGO-induced increases and Gly-Low–mediated prevention in PWV, the *ex vivo* experiments were conducted only in males. Future studies should therefore investigate the molecular effects of MGO and Gly-Low in females as well. Together, these limitations highlight the need for future studies using integrated longitudinal designs, additional AGE precursors, direct compliance testing, and refined monitoring approaches to further clarify the role of glycation stress and the therapeutic potential of Gly-Low in vascular aging.

## Supplementary Material

Supplementary Materials

Supplementary Figures

Supplementary Table 1
