# Supplementary material for: Methylglyoxal-induced glycation stress promotes aortic stiffening: putative mechanistic roles of oxidative stress and cellular senescence
Source: Aging (Albany NY). 2025 Nov 14;17(11):2717–43. doi: 10.18632/aging.206335 (PMC12705182; doi:10.18632/aging.206335)
Supplement: Supplementary Figures [file aging-17-11-206335-s002.pdf]

SUPPLEMENTARY FIGURES

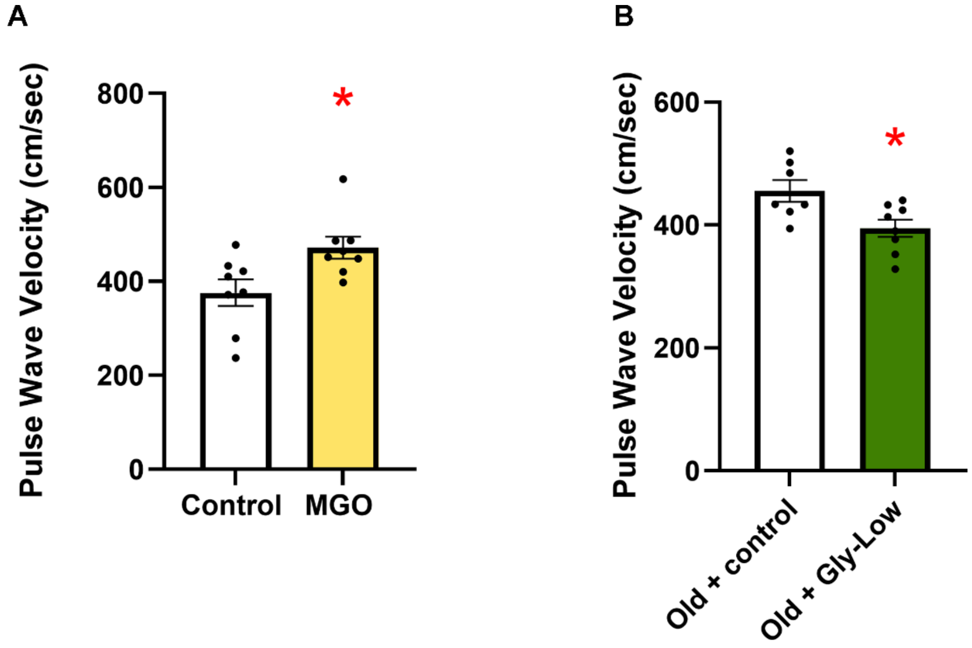

Supplementary Figure 1. MGO-induced glycation stress increases aortic stiffening in young female mice and Gly-Low supplementation lowers aortic stiffness in old female mice. (**A**) Aortic PWV measured in young (3 months) female mice after 2-month intervention with MGO in drinking water (n=8/group). (**B**) Aortic PWV measured in old (20 months) female mice after 4-month supplementation with Gly-Low. All data were analyzed using unpaired t-tests. All values are in mean ± SEM, *p<0.05 vs. control.

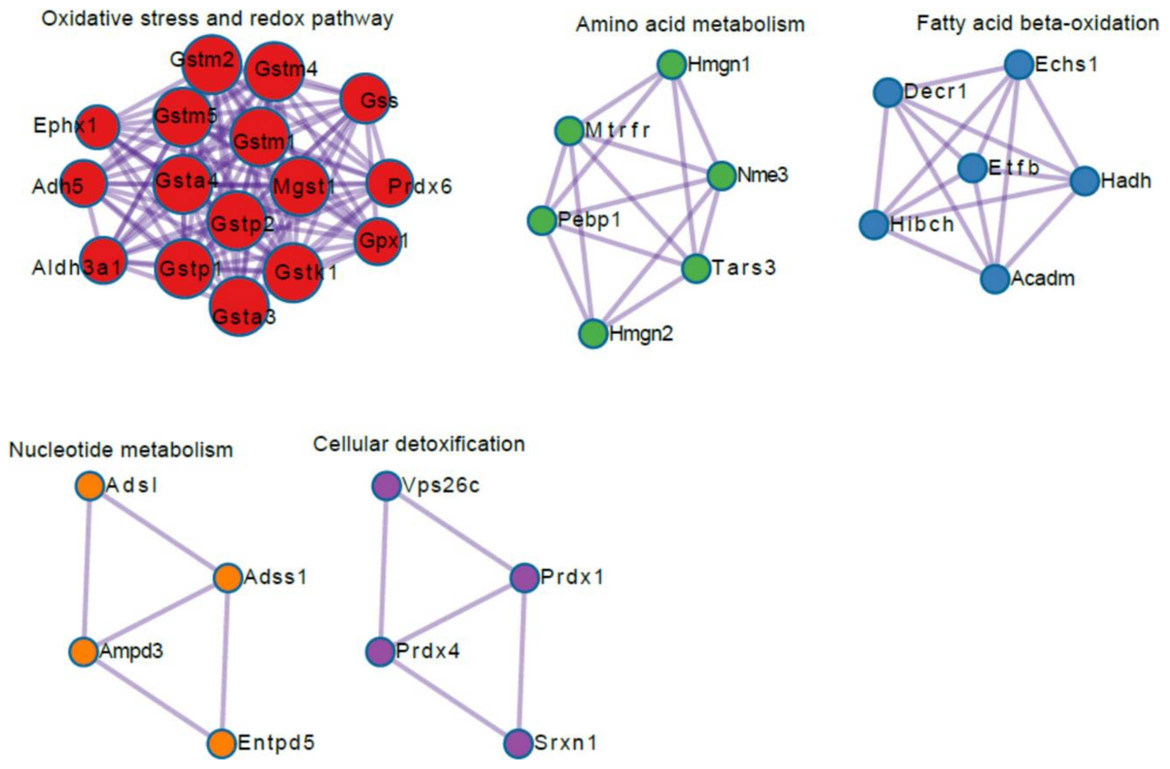

Supplementary Figure 2. MCODE-derived network modules among Gly-Low-responsive genes. Protein interaction network was constructed from the 280 genes significantly upregulated by Gly-Low treatment in aortic rings (fold-change ≥ 1.5, FDR < 0.05) in the absence and presence of MGH-1. The network was clustered using the MCODE algorithm (degree cutoff = 2, node score cutoff = 0.2, K-core = 2, max depth = 100), yielding the high-confidence modules (MCOD score > 3)

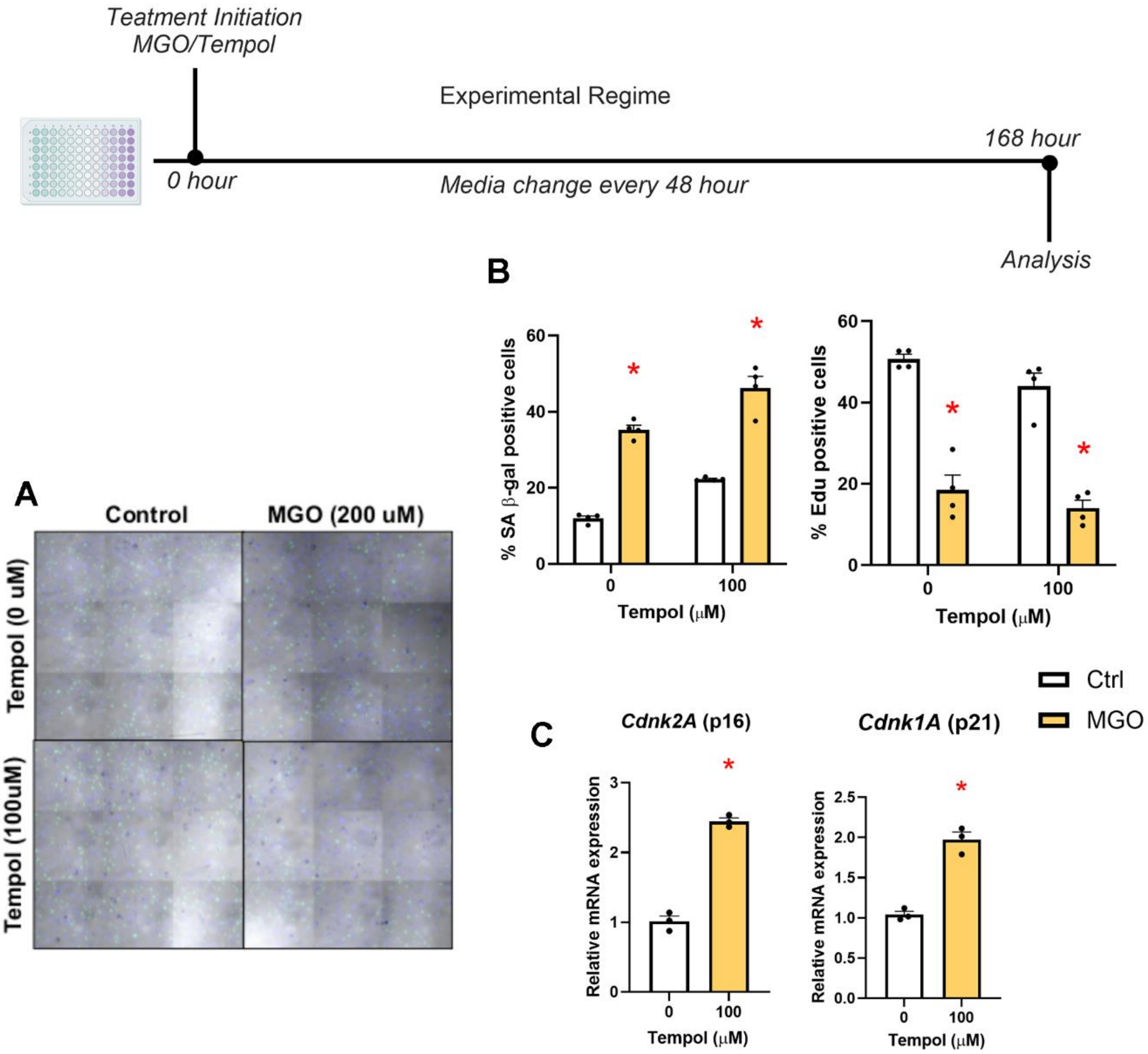

Supplementary Figure 3. MGO induces cellular senescence in endothelial cells independent of ROS levels. (**A**) Senescence-associated β-galactosidase and EdU staining for HAECs treated with MGO/TEMPOL. (**B**) Quantitative analysis of SA-β-gal and EdU staining. (**C**) mRNA expression of various senescence-associated genes. All data were analyzed using unpaired t-tests, as comparisons were made within each group (absence or presence of TEMPOL). All values are in mean ± SEM, *p<0.05 vs. control of same condition.

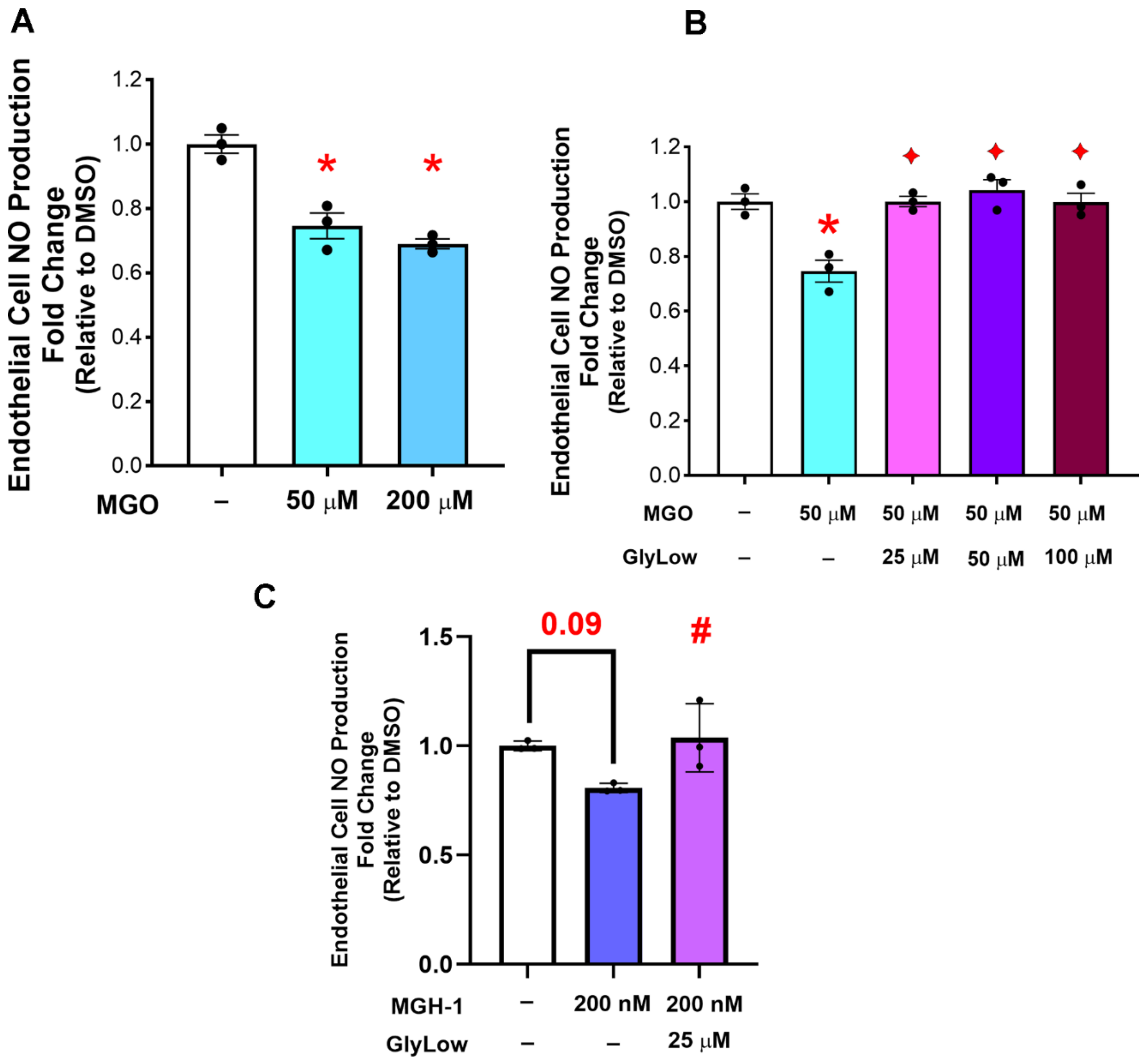

Supplementary Figure 4. MGO and MGH-1 induce endothelial dysfunction by lowering nitric oxide (NO) levels. (**A**) HAECs NO production when incubated in standard media vs. 50/200 µM MGO (n=3/group). (**B**) NO production when incubated with MGO in the presence of 25, 50, and 100 µM Gly-Low (n=3/group). (**C**) NO production when incubated with 200 nM MGH-1 in the presence/absence of 25 µM Gly-Low. Statistical analysis was performed using one-way ANOVA. All values are in mean ± SEM, *p<0.05 vs. control; ◆p<0.05 vs. MGO alone; #p<0.05 vs. MGH-1 alone.
