# Supplementary material for: Methylglyoxal-induced glycation stress promotes aortic stiffening: putative mechanistic roles of oxidative stress and cellular senescence
Source: Aging (Albany NY). 2025 Nov 14;17(11):2717–43. doi: 10.18632/aging.206335 (PMC12705182; doi:10.18632/aging.206335)
Supplement: Supplementary Table 1 [file aging-17-11-206335-s003.pdf]

SUPPLEMENTARY TABLE

Supplementary Table 1. Diameter and wall thickness of intervention-naive aorta rings after 48-hour incubation in Control (Standard media), MGO, and Gly-Low.

	Control (N=5)	MGO (N=5)	MGO+Gly-Low (N=5)
Aortic wall thickness (uM)	53.4 ± 1.3	53.7 ± 2.4	56.7 ± 4.3
Aortic diameter (uM)	618.74 ± 13.7	647 ± 22.2	653.78 ± 36.8

Data reported as Mean ± SEM.
